# Glucagon Prevents Cytotoxicity Induced by Methylglyoxal in a Rat Neuronal Cell Line Model

**DOI:** 10.3390/biom11020287

**Published:** 2021-02-15

**Authors:** Mohammad Sarif Mohiuddin, Tatsuhito Himeno, Yuichiro Yamada, Yoshiaki Morishita, Masaki Kondo, Shin Tsunekawa, Yoshiro Kato, Jiro Nakamura, Hideki Kamiya

**Affiliations:** Department of Internal Medicine, Division of Diabetes, Aichi Medical University School of Medicine, Nagakute 480-1195, Japan; sarif@aichi-med-u.ac.jp (M.S.M.); thimeno@aichi-med-u.ac.jp (T.H.); yamada.yuuichirou.306@mail.aichi-med-u.ac.jp (Y.Y.); morishita.yoshiaki.517@mail.aichi-med-u.ac.jp (Y.M.); kondou.masaki.330@mail.aichi-med-u.ac.jp (M.K.); tsune87@aichi-med-u.ac.jp (S.T.); ykato4@aichi-med-u.ac.jp (Y.K.); jiro@aichi-med-u.ac.jp (J.N.)

**Keywords:** diabetic polyneuropathy, glucagon, methylglyoxal, peripheral neuronal cell, 50B11, apoptosis, PKA, cAMP

## Abstract

Although diabetic polyneuropathy (DPN) is a frequent diabetic complication, no effective therapeutic approach has been established. Glucagon is a crucial hormone for glucose homeostasis but has pleiotropic effects, including neuroprotective effects in the central nervous system. However, the importance of glucagon in the peripheral nervous system (PNS) has not been clarified. Here, we hypothesized that glucagon might have a neuroprotective function in the PNS. The immortalized rat dorsal root ganglion (DRG) neuronal cell line 50B11 was treated with methylglyoxal (MG) to mimic an in vitro DPN model. The cells were cultured with or without glucagon or MG. Neurotoxicity, survival, apoptosis, neurite projection, cyclic adenosine monophosphate (cAMP), and protein kinase A (PKA) were examined. Glucagon had no cytotoxicity and rescued the cells from neurotoxicity. Cell survival was increased by glucagon. The ratio of apoptotic cells, which was increased by MG, was reduced by glucagon. Neurite outgrowth was accelerated in glucagon-treated cells. Cyclic AMP and PKA accumulated in the cells after glucagon stimulation. In conclusion, glucagon protected the DRG neuronal cells from MG-induced cellular stress. The cAMP/PKA pathway may have significant roles in those protective effects of glucagon. Glucagon may be a potential target for the treatment of DPN.

## 1. Introduction

Diabetic polyneuropathy (DPN) is a chronic diabetic complication. DPN causes diabetic feet, including foot infections, ulcers, and limb amputations. Intensive blood glucose control has been proven to prevent the development of DPN in type 1 diabetes [1,2]. Additionally, etiology-oriented therapeutic approaches to DPN have been proposed and used in limited countries: an aldose reductase inhibitor epalrestat in Japan and India [3] and alpha-lipoic acid and benfotiamine in Germany [4,5]. However, no approach has yet achieved sufficient efficacies in DPN.

Glucagon is a 29 amino acid peptide secreted from alpha cells in the pancreas and is responsible for glucose homeostasis by glucose efflux from the liver [6]. Other than gluconeogenesis, glucagon has pleiotropic effects: cardioprotection [7,8] and reduction of obesity [9]. Regarding the nervous systems, it has been reported that in post-traumatic brain injury, glucagon produced a significant neuroprotective effect [10]. Recently, Li et al. reported that a triagonist of glucagon-like peptide-1(GLP-1)/gastric inhibitory polypeptide/glucagon receptors produced neurotrophic and neuroprotective action by reducing cell cytotoxicity and glutamate excitotoxicity by elevating cyclic adenosine monophosphate (cAMP) levels in human neuroblastoma cell-line SH-SY5Y [11]. However, no previous study has reported the neuroprotective effect of glucagon in the peripheral nervous system. We previously reported that deficiency of glucagon gene-derived peptides, including GLP-1 and glucagon, caused peripheral neuropathy in mice [12]. Additionally, we have revealed that GLP-1 receptor agonists showed neuroprotective effects in an in vitro model of oxidative insult and an in vivo model of DPN [13,14]. Here, we investigated the neuroprotective effects of glucagon on the dorsal root ganglion (DRG) neuronal cells.

To investigate the effects of glucagon at the cellular level, we utilized cellular stress induced by methylglyoxal (MG). Several factors are suggested to be responsible for DPN, including aldose reductase activation [15,16], deposition of advanced glycation end-products (AGEs) [17], oxidative stress [18,19,20,21], increased release of inflammatory mediators [22,23], and lack of neurotrophic factors [24,25,26]. MG is a significant source of intracellular AGEs and causes cytotoxicity [27], apoptosis [28], mitochondrial reactive oxygen species (ROS) production [29,30], and reduction of cellular viability [31]. It was reported by Beatrice et al. that MG (250–750 μM) reduced cellular viability, transient accumulation of intracellular [Ca^2+^]_i_, and neurite outgrowth in mouse DRG neurons [32]. Under normal physiological conditions, MG is formed as a byproduct of glycolysis, which is usually detoxified by various systems, mainly the glyoxalase system. However, in hyperglycemic conditions such as diabetes, the formation of MG has been found to be accelerated [29,33]. Plasma MG concentration is elevated in patients with poorly controlled type 2 diabetes compared to those of healthy persons [34,35]. Accumulating evidence has suggested that a high MG level is the key factor for developing DPN [36]. A study showed that in streptozotocin-induced diabetic mice, a high level of MG in the sciatic nerve has been found [37]. Patients with DPN showed a reduction in glyoxalase activity when compared to patients with diabetes [38]. It was reported that MG-derived AGEs such as hydroimidazolones of MG were associated with the progression of DPN with type 1 diabetes [39]. Hence, we hypothesized that MG could become a key regulator to reproduce DPN conditions in vitro. Therefore, in this study, we have used the immortalized DRG neuronal cells treated by MG to assume the effect of glucagon on the peripheral nervous system in patients with diabetes.

## 2. Materials and Methods 

Until declaration, all reagents and materials were purchased from Thermo Fisher Scientific (Waltham, MA, USA).

### 2.1. Cell Culture 

The DRG neuronal cell line (50B11) was established and kindly provided by Dr. A. Höke (Johns Hopkins University, Baltimore, MD, USA) [40]. The cells were maintained at 37 °C under 5% carbon dioxide in the maintaining media (MM) consisting of Neurobasal™ medium supplemented with 10% fetal bovine serum (FBS), 2 mmol/L L-glutamine, and B-27 supplement^TM^. For each experiment, cells were cultured in the treatment medium (TM) consisting of Dulbecco’s Modified Eagle Medium (DMEM) (Cat: 11965-092, 40 mmol/L glucose) supplemented with 5% FBS. In TM, the cells were treated with or without glucagon (1 or 100 pmol/L) or forskolin, an activator of adenylate cyclase, (10 or 25 μmol/L). Cellular stress was produced by MG (0.1 or 0.5 mmol/L). The stock solution of 1 mM glucagon was made by resolving glucagon in 0.1 N HCl. For the control condition in each experiment, the same amount of 0.1 N HCl without glucagon was supplemented.

### 2.2. Cytotoxicity Measurement 

Cells were seeded into 96-well plates at a density of 1 × 10^4^ cells/well in 100μL of TM and incubated for 24 h. After 6 h of treatments with glucagon or forskolin in the presence or absence of MG, cytotoxicity was measured using a lactate dehydrogenase (LDH) assay (Cytotoxicity LDH Assay Kit-WST, Dojindo Laboratories, Mashiki, Japan) according to the manufacturer’s instructions. Optical density (OD) was measured by determining the absorbance at 490 nm using a microplate reader (SpectraMax M5, Molecular Devices, Sunnyvale, CA, USA). The lysis buffer served by the manufacturer revealed maximum LDH release, which was used as high control. Low control was a condition without any treatment. Cytotoxicity was calculated by the following formula: cytotoxicity (%) = (sample OD − low control OD)/(high control OD − low control OD) × 100. All OD values were used after subtraction of the background value from each OD value.

### 2.3. Cell Survival Assay

Cell survivalwas evaluated using a CellTiter96™ AQueous One Solution Cell Proliferation assay (Promega Corporation, Madison, WI, USA) which employed 3-(4,5-dimethylthiazol-2-yl)-5-(3-carboxymethoxyphenyl)-2-(4-sulfophenyl)-2H-tetrazolium (MTS) according to the manufacturer’s protocol. Cells were seeded into 96-well plates at a density of 1 × 10^4^ cells/well in 100 μL TM. After 24 h, the cells were treated with or without MG in the presence or absence of glucagon. After 6 h of treatment, cell survival was determined using absorbance at 490 nm, which was measured on a microplate reader (SpectraMax M5). The following formula measured survival: cell survival (%) = (sample OD/control OD) × 100. Each OD value was used after subtraction of the background value from each OD value. 

### 2.4. Apoptosis Estimation

Cells were seeded in a 24-well plate at a density of 5 × 10^4^ cells/well in 500 μL TM. After 24 h of incubation, apoptosis was induced by 0.5 mmol/L of MG. The degree of apoptosis was measured by using an APOPercentage^TM^ assay (Biocolor, Belfast, Northern Ireland, UK) according to the manufacturer’s instructions. The assay is a dye uptake assay, which stains only apoptotic cells with a purple dye. Apoptosis was assessed after 6 h of exposure to MG with or without glucagon. The cells were gently washed twice with 500 μL of phosphate-buffered saline (PBS) per well to remove the non-cell bound dye and fixed with 2% paraformaldehyde (PFA). To count the total number of cells, cells were counterstained with 4′,6-diamidino-2-phenylindole (DAPI). Photographs were taken by using a charge-coupled device (CCD) camera-equipped microscope (IX73, Olympus Corporation, Tokyo, Japan). The percentage of apoptosis was measured by the number of apoptotic cells divided by the total number of cells.

### 2.5. Mitochondrial ROS Measurement

Mitochondrial ROS was assessed using MitoSOX^TM^ Red mitochondrial superoxide indicator [41]. Cells were seeded in a 24-well plate at a density of 3 × 10^4^ cells/well in TM. After 6 h of treatment with glucagon or 25 μmol/L forskolin in the presence or absence of MG, cells were washed with PBS. The cells were exposed to 5 μmol/L of MitoSOX^TM^ Red for 10 min at 37 °C. After another washing with PBS, cells were counterstained with DAPI. The fluorescence signal was observed using an IX73 inverted microscope (Olympus Corporation). 

### 2.6. Neurite Outgrowth 

The cells were seeded in a 6-well plate at a density of 1 × 10^5^ cells/well in TM. Neurite outgrowth was checked after 24 h of treatment with glucagon or 25 µmol/L forskolin. The cells were fixed with 2% PFA for 10 min at 4 °C. After the fixation, photographs were taken using a CCD camera-equipped microscope (IX73, Olympus Corporation). The cells with neurite outgrowth were defined as the cells with neurite projection which is equal to or longer than the length of the cell body [42]. The percentage of cells with neurites was calculated by the number of cells with neurites divided by the total number of cells.

### 2.7. cAMP Measurement 

Concentration of intracellular cAMP was measured using an enzyme immunoassay kit (Cayman Chemical, Ann Arbor, MI, USA) [43,44]. The cells were seeded in a 6-well plate at a density of 5 × 10^5^ cells/well in MM. After 24 h, the cells were treated with or without glucagon or 10 µmol/L of forskolin for 20 min. The cells were collected from the wells by scraping after 20 min of treatment with 0.1 mol/L hydrochloric acid on ice. The supernatant was collected after centrifugation and a cAMP assay was performed according to the manufacturer’s instructions. 

### 2.8. Protein Kinase A (PKA) Activity Detection

To quantify the activity of cAMP-dependent PKA [45], a PKA Colorimetric Activity kit (Thermo Fisher Scientific, Waltham, MA, USA) was used. The cells were seeded in a 6-well plate at a density of 5 × 10^5^ cells/well in MM. After 24 h, the cells were treated with or without glucagon in the presence or absence of 10 µmol/L H89, PKA inhibitor [46], in MM. After 30 min, the cells were incubated with a cell lysis buffer supplied by the manufacturer for another 30 min on ice with occasional vortexing. After 10 min of centrifugation, the supernatant was collected and used to perform a PKA assay according to the manufacturer’s instructions.

### 2.9. Statistical Analysis

All data are presented as mean ± standard deviation (SD). All data was produced in at least three individual and separate experiments. Student’s t-test and one-way analysis of variance, followed by Bonferroni’s test, were performed using IBM SPSS statistics 20 (Armonk, NY, USA). A p-value of less than 0.05 was considered statistically significant. 

## 3. Results

### 3.1. Glucagon Decreased Cytotoxicity Induced by MG

**Figure 1:**
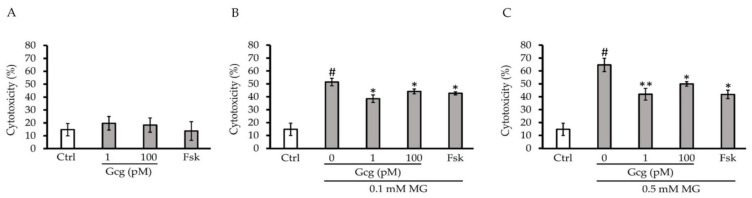
Cytotoxicity assay: (**A**) After 6 h of treatment with glucagon or forskolin, no significant cytotoxicity was identified. (**B**,**C**) Although 0.1 and 0.5 mmol/L methylglyoxal (MG) exhibited significant cytotoxicity in the cells, glucagon (1, 100 pmol/L) and forskolin (25 µmol/L) significantly attenuated cytotoxicity. # *p* < 0.001 versus control without MG; ** *p* < 0.005 versus control, * *p* < 0.05 versus control. Ctrl: control, Gcg: glucagon, MG: methylglyoxal, Fsk: forskolin, mM: mmol/L, pM: pmol/L.

After 6 h of treatment, there was no cytotoxicity produced by glucagon or forskolin (control 14.8 ± 4.8%, 1 pmol/L glucagon 19.7 ± 5.3, *p* = 0.39 versus control, 100 pmol/L glucagon 18.3 ± 5.5, *p* = 0.53, 25 µmol/L forskolin 13.7 ± 7.3, *p* = 0.87; *n* = 3 in each group) (Figure 1A). Although 0.1 and 0.5 mmol/L of MG exhibited significant cytotoxicity in the cells, glucagon and forskolin significantly attenuated the cytotoxicity (0.1 mmol/L MG: control 51.5 ± 2.9%, *p* < 0.005 versus no treatment with MG or other agents, 1 pmol/L glucagon 38.5 ± 2.9, *p* < 0.05 versus control, 100 pmol/L glucagon 44.2 ± 1.9, *p* < 0.05, 25 µmol/L forskolin 42.7 ± 1.2, *p* < 0.05; 0.5 mmol/L MG: control 64.7 ± 5.2%, *p* < 0.001 versus no treatment with MG or other agents, 1 pmol/L glucagon 42.0 ± 4.6, *p* < 0.01 versus control, 100 pmol/L glucagon 50.0 ± 1.8, *p* < 0.05, 25 µmol/L forskolin 41.8 ± 3.3, *p* < 0.05; *n* = 3 in each group) (Figure 1B,C).

### 3.2. Survival of DRG Neuronal Cells Was Promoted by Glucagon

**Figure 2:**
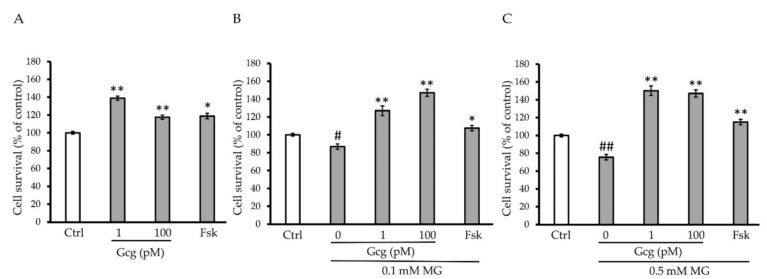
Cell survival assay: (**A**–**C**) MG significantly reduced the survival of dorsal root ganglion (DRG) neuronal cells. Glucagon and forskolin increased the cell survival which was reduced by MG. # *p* < 0.05 versus control without MG, glucagon, or forskolin; ## *p* < 0.001 versus control without MG, glucagon, or forskolin; * *p* < 0.01 and ** *p* < 0.001 versus control. *n* = 3 in each group. Error bars: standard deviation. Ctrl: control, Gcg: glucagon, MG: methylglyoxal, Fsk: forskolin, mM: mmol/L, pM: pmol/L.

The survival of DRG neuronal cells was decreased by MG (no MG 100.0 ± 1.5%; 0.1 mmol/L MG 86.8 ± 3.1, *p* < 0.05 versus no MG; 0.5 mmol/L MG 75.5 ± 1.5, *p* < 0.001; *n* = 3 in each group) (Figure 2A–C). However, glucagon and forskolin had the capability to improve the decrease in survival (no MG: 1 pmol/L glucagon 138.9 ± 2.3, *p* < 0.001 versus no glucagon, 100 pmol/L glucagon 117.6 ± 2.4, *p* < 0.001, 25 µmol/L forskolin 118.8 ± 3.0, *p* < 0.01; 0.1 mmol/L MG: 1 pmol/L glucagon 127.0 ± 5.4, *p* < 0.001 versus no glucagon, 100 pmol/L glucagon 147.0 ± 4.1, *p* < 0.001, 25 µmol/L forskolin 107.0 ± 3.1, *p* < 0.01; 0.5 mmol/L MG: 1 pmol/L glucagon 150.2 ± 3.2, *p* < 0.001 versus no glucagon, 100 pmol/L glucagon 147.0 ± 2.7, *p* < 0.001, 25 µmol/L forskolin 115.1 ± 3.1, *p* < 0.001; *n* = 3 in each group) (Figure 2B,C). 

### 3.3. Glucagon Attenuated MG-Induced Apoptosis 

**Figure 3:**
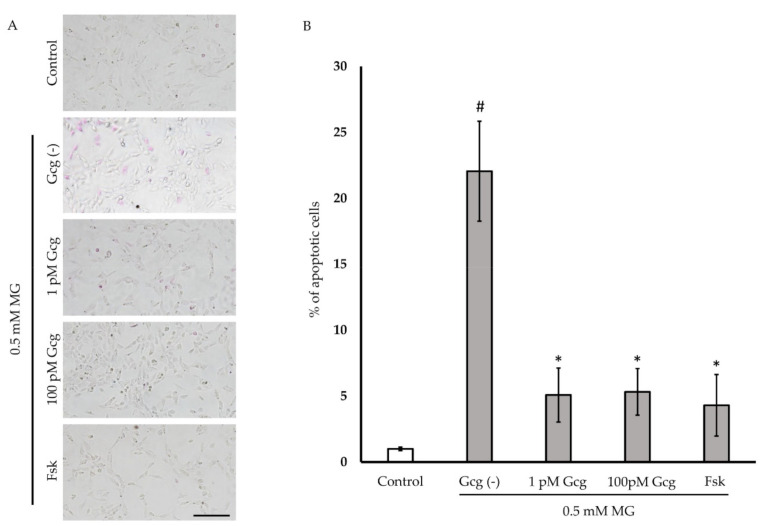
Apoptosis estimation: (**A**) Apoptotic cells were stained with purple dye. Many apoptotic cells were observed in the cells treated with 0.5 mM MG (control). However, the number of apoptotic cells was low in cells treated with glucagon or forskolin. Scale bar: 200 µm. (**B**) The percentage of apoptosis was significantly reduced in the cells treated with glucagon or forskolin. # *p* < 0.05 versus MG (−); * *p* < 0.05 versus MG treatment without glucagon or forskolin (control). *n* = 3 in each group. Error bars: standard deviation. Gcg: glucagon, MG: methylglyoxal, Fsk: forskolin, pM: pmol/L, mM: mmol/L.

Six hours after treatment with 0.5 mmol/L of MG, more than 20% of the neuronal cells exhibited apoptosis (Figure 3A,B). The percentage of apoptotic cells was reduced in the cells treated with glucagon or forskolin (no MG 1.0 ± 0.1%; 0.5 mmol/L MG 22.1 ± 3.8, *p* < 0.001 versus no MG; 1 pmol/L glucagon 5.1 ± 2.1, *p* < 0.05 versus group with glucagon (−)/MG (+); 100 pmol/L glucagon 5.3 ± 1.8, *p* < 0.05; 25 µmol/L forskolin 4.3 ± 2.3, *p* < 0.05; *n* = 3 in each group). 

### 3.4. Mitochondrial ROS Production Induced by MG Was Inhibited by Glucagon

**Figure 4:**
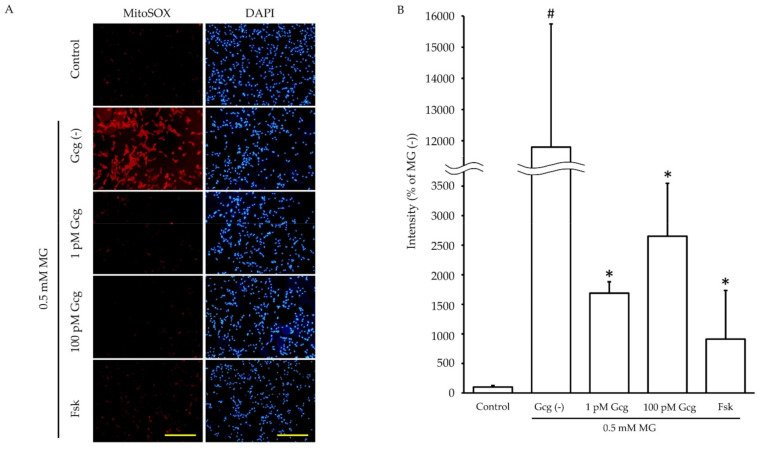
Mitochondrial reactive oxygen species (ROS) measurement: (**A**) The fluorescence images of MitoSOX™ (red) showed mitochondrial ROS production of neuronal cells with or without glucagon or forskolin in the presence or absence of MG. Blue: DAPI (nuclei). Scale bar: 200 μm. (**B**) The fluorescence intensity quantified by ImageJ software. The production of ROS by MG was significantly reduced in the cells treated with glucagon or forskolin. # *p* < 0.05 versus MG (−); * *p* < 0.05 versus control with MG; *n* = 3 in each group. Error bars: standard deviation, Gcg: glucagon, MG: methylglyoxal, Fsk: forskolin, mM: mmol/L, pM: pmol/L.

Incubation for 6 h with MG promoted mitochondrial ROS production in DRG neuronal cells (no MG 100.0 ± 27.2%; 0.5 mmol/L MG 11795.7 ± 3948.2, *p* < 0.05) (Figure 4). However, the increase in ROS production induced by MG was inhibited by glucagon or forskolin (1 pmol/L glucagon 1687.9 ± 193.7, *p* < 0.05 versus no glucagon with MG, 100 pmol/L glucagon 2651.8 ± 894.2, *p* < 0.05, 25 µmol/L forskolin 912.5 ± 822.4, *p* < 0.05).

### 3.5. Neurite Projection Was Increased by Glucagon 

**Figure 5:**
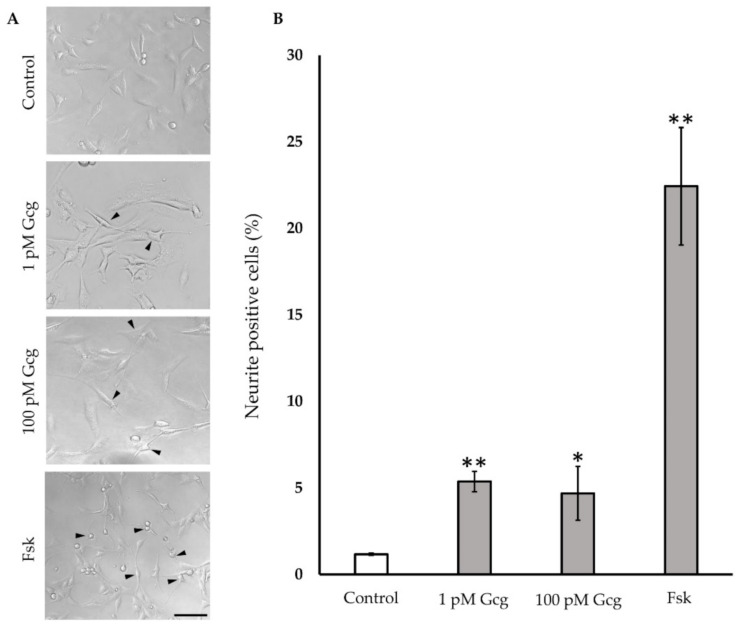
Neurite outgrowth: (**A**) Light microscopic photography showed the neurite projection in DRG neuronal cells with or without glucagon and forskolin. Arrowheads indicate cells with neurite. Scale bar: 100 μm. (**B**) The percentage of neurite positive neurons was higher in the cells treated with glucagon and forskolin. ** *p* < 0.001 versus control; * *p* < 0.05 versus control; *n* = 3 in each condition; Error bars: standard deviation. Gcg: glucagon, MG: methylglyoxal, Fsk: forskolin, pM: pmol/L.

After a 24-h treatment of neuronal cells with glucagon or forskolin, neuronal outgrowth was evaluated (Figure 5). Glucagon and forskolin significantly promoted neuronal outgrowth; no neurite projection was found in the cells without treatment (control 1.18 ± 0.06%; 1 pmol/L glucagon 5.4 ± 0.6%, *p* < 0.001 versus control, 100 pmol/L glucagon 4.7 ± 1.6, *p* < 0.05, 25 μmol/L forskolin 22.4 ± 3.4, *p* < 0.001).

### 3.6. Production of Cyclic Adenosine Monophosphate (cAMP) Increased by Glucagon Stimulation

**Figure 6:**
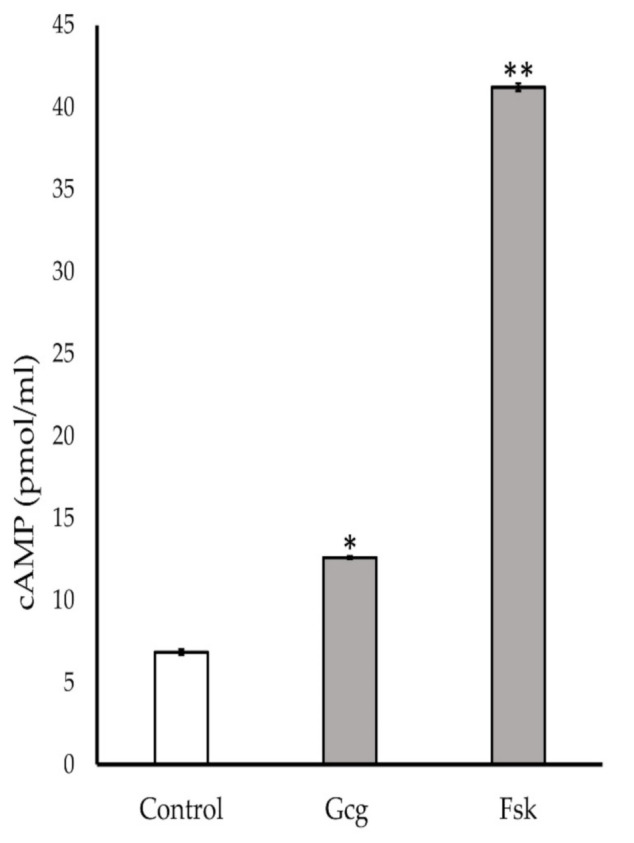
Cyclic adenosine monophosphate (cAMP) assay: Accumulation of cAMP was found in the cells treated with 1 pmol/L glucagon or 10 μmol/L forskolin. * *p* < 0.05 versus control, ** *p* < 0.001 versus control; Error bars: standard deviation. Gcg: glucagon, Fsk: forskolin.

The neuronal cells were treated with MM for 20 min with glucagon or forskolin. Glucagon and forskolin showed significant production of cAMP (control 6.9 ± 0.2 pmol/mL; 1 pmol/L glucagon 12.6 ± 0.1, *p* < 0.05 versus control, 10 µmol/L forskolin 41.2 ± 0.2, *p* < 0.001) (Figure 6).

### 3.7. PKA Activity Was Increased by Glucagon

**Figure 7:**
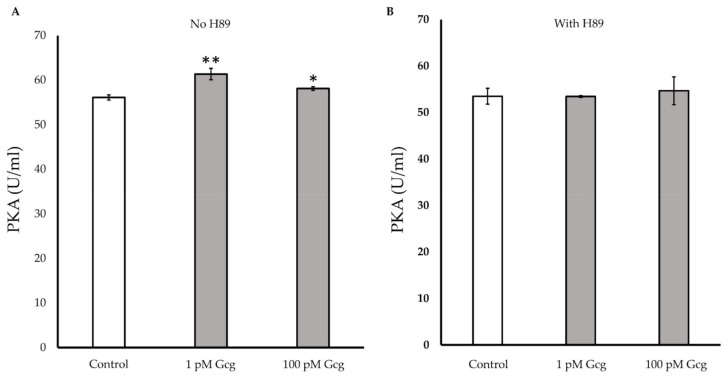
Protein kinase A (PKA) detection: (**A**) The activity of PKA significantly increased in the cells treated with 1 or 100 pmol/L of glucagon. (**B**) When the cells were treated with glucagon in the presence of the PKA inhibitor H89, no significant increase in PKA was observed. * *p* < 0.05 versus control, ** *p* < 0.01 versus control, Error bars: standard deviation. Gcg: glucagon, Fsk: forskolin, pM: pmol/L.

After 30 min of treatments with 1 or 100 pmol/L of glucagon, the glucagon significantly increased intracellular PKA activity (control 56.1 ± 0.6 U/mL; 1 pmol/L glucagon 61.4 ± 1.3, *p* < 0.01 versus control, 100 pmol/L glucagon 58.1 ± 0.4, *p* < 0.05) (Figure 7A). When the cells were treated with glucagon in the presence of the PKA inhibitor, no significant increase in PKA was observed (control 53.5 ± 1.7; 1 pmol/L glucagon 53.5 ± 0.2, *p* = 0.97 versus control, 100 pmol/L glucagon 54.7 ± 3.0, *p* = 0.65) (Figure 7B). 

## 4. Discussion

In this study, we have revealed the neuroprotective functions of glucagon in peripheral neuronal cells for the first time. First, we have investigated the cytoprotective effects of glucagon in the neuronal cell-line 50B11 from the cell stress induced by MG. The results show that glucagon reduced neurotoxicity, apoptosis, mitochondrial ROS production, and promoted neuronal survival. Second, we verified the regulatory roles of glucagon in cellular biology; glucagon promoted neuronal elongation and increased intracellular cAMP and PKA activities. When investigating the roles of glucagon, even in physiological concentrations of glucagon, neuronal cells suffered no significant cytotoxicity. Furthermore, glucagon increased cell survival. Although one previous study indicated direct cytoprotective effects of glucagon in hepatocytes [47], the current study is the first report of cytoprotective effects in neuronal cells. To examine further cytoprotective potential of glucagon, experiments using the cell stress model were performed. As a result, cytoprotective effects were proven in a cytotoxicity assay, cell survival assay, and apoptosis assay. Additionally, mitochondrial oxidative stress was decreased by the supplementation of glucagon. These results clearly indicate the beneficial effects of glucagon, even in stressed circumstances, on DRG neuronal cells. To clarify the regulatory mechanisms of these effects in-depth, the intracellular signaling cAMP/PKA pathway was examined. The cAMP/PKA pathway has been verified in hepatocytes to be an intracellular signaling pathway activated by glucagon stimulation [48]. Regarding the nervous system, it has been reported in the central nervous system that activation of cAMP/PKA pathway increased synaptic plasticity [49], enhanced neurotransmitter release [50], and reduced cellular vulnerability to oxidative stress in astrocytes [51]. Additionally, it was reported that increase of cAMP promoted the regeneration of DRG axons in a mice model of sciatic nerve injury [52]. Therefore, we evaluated the association of cAMP and PKA in a cell line treated with glucagon. Treatment with physiological concentrations of glucagon successfully promoted accumulation of cAMP and activation of PKA in the cells. Additionally, neurite outgrowths, which have been proven to be enhanced by cAMP analogs in peripheral neuronal cells [42], were promoted by glucagon. This is the first study that identified the activation of the cAMP/PKA pathway induced by glucagon in DRG neuronal cells. Although the activation of the cAMP/PKA pathway might produce the neuroprotective effects of glucagon, the current study includes no experiment in which cAMP/PKA activation was examined during the exposition to the cellular stress by MG. Therefore, further research to elucidate the intracellular mechanisms in the neuroprotective effects of glucagon should be performed in the future.

There are four limitations in the current study. First, this cell line is immortalized; although neurons in vivo rarely proliferate, the neuronal cells rapidly proliferate. Therefore, the current results, especially cytotoxicity and cell survival, may be altered in DRG neurons in vivo. To overcome this limitation, further research using primary cultures of DRG or in vivo animal studies should be considered in the future. Second, a single stressor, MG, was used for a short duration in this study. However, DPN is a chronic diabetic complication whose pathogenesis consists of multiple factors. Although we have struggled to improve the in vitro model of DPN using longer durations of treatment with lower concentrations of MG, inconsistent results were observed. Combinations with other stressors may improve this drawback. Third, we have verified the effects of glucagon using an in vitro model. As glucagon increases the blood glucose level, chronic administration of glucagon as a treatment of DPN may worsen glycemic condition [53]. Therefore, to realize the administration of glucagon in the treatment of DPN, it is necessary to develop technology to overcome the issue, e.g., the development of tissue-specific agonists and drug delivery systems. Fourth, the changes of PKA activity were moderate. The activity of PKA changed only ~5% between conditions with or without glucagon. Furthermore, the PKA inhibitor H89 barely inhibited the activities. The mild changes might be caused by the shortage of exposure time to H89. Further experimental efforts should be performed in the future. 

## 5. Conclusions

In conclusion, we have successfully verified novel neuroprotective actions of glucagon against MG-induced cellular stress in peripheral neuronal cells. Our report may support identifying the pharmacological effects of glucagon in neurons for future study and opens a hidden era to study glucagon for future researchers.

## Data Availability

Not applicable.
